# Engagement, Disengagement and Re‐Engagement in Mental Health Services Among Young Patients With First‐Episode Psychosis: A Scoping Review

**DOI:** 10.1111/eip.70100

**Published:** 2025-10-09

**Authors:** Joyce Mlay, Saeeda Paruk, Andrew Tomita, Richard Lessells

**Affiliations:** ^1^ Discipline of Public Health Medicine, School of Nursing and Public Health University of KwaZulu‐Natal Durban South Africa; ^2^ Health Economics and HIV and AIDS Research Division (HEARD) University of KwaZulu‐Natal Durban South Africa; ^3^ Discipline of Psychiatry, Nelson R Mandela School of Medicine University of KwaZulu‐Natal Durban South Africa; ^4^ Centre for Rural Health, School of Nursing and Public Health University of KwaZulu‐Natal Durban South Africa; ^5^ KwaZulu‐Natal Research Innovation and Sequencing Platform (KRISP), College of Health Sciences University of KwaZulu‐Natal Durban South Africa; ^6^ Centre for the AIDS Programme of Research in South Africa (CAPRISA) Durban South Africa

**Keywords:** disengagement, engagement, FEP, follow‐up, re‐engagement

## Abstract

**Introduction:**

Engagement in mental health services among young patients with first‐episode psychosis (FEP) is crucial for preventing relapse. We conducted this scoping review to establish the proportion and determinants of engagement, disengagement and re‐engagement in outpatient mental health care among young patients from 13 to 35 years with FEP.

**Methods:**

We used the guidelines for scoping review by the Joanna Briggs Institute (JBI) and the Arksey and O'Malley framework. We searched for published and unpublished studies guided by the inclusion criteria: studies published in English from 1990, the expansion of psychiatric outpatient, focused on engaged, disengaged and re‐engaged in outpatient mental health care using quantitative, qualitative and mixed methods studies. We performed the numerical and thematic analysis and reporting using Preferred Reporting Items for Systematic Reviews and Meta‐Analysis Extension for scoping review (PRISMA‐ScR).

**Results:**

About 25 articles published from 2002 to 2020 met the inclusion criteria for this review; the proportion of young people with FEP who remained engaged was 45.5% for 6 months of follow‐up, and the proportion of re‐engagement after initial disengagement was 78.8%. Disengagement ranged between 13% and 56.3% for 12–36 months. The socio‐demographic factors associated with disengagement were older age, male, black, unmarried status, living alone, unemployment, social and material deprivation, poverty, substance use and involvement with criminal justice. Some clinical determinants included a history of mental illness in the family and a psychosis disorder diagnosis other than schizophrenia.

**Conclusion:**

Disengagement from mental health services is consistently high among people with FEP, indicating the need for intervention studies that address the associated individual and clinical factors to ensure their retention in treatment.

## 1 Introduction

First‐episode psychosis (FEP) is the first time a person shows signs of losing contact with reality, usually occurring in adolescence/young adulthood. As it affects the way a person thinks and behaves, it can severely impair everyday functioning when left untreated (Breitborde et al. 2009; Menezes et al. 2006; Petersen et al. 2012). Engaging in care during this critical period is essential for achieving favourable long‐term outcomes (Becker et al. 2016). Failure to engage in care can result in significant negative consequences, including an increased risk of relapse, reduced quality of life and impaired social functioning (Stewart 2013). This is especially concerning for young people with psychotic disorders, as outcome trajectories are established during the early stages of the disorder, and the majority of young adults are particularly at high risk for disengaging from mental health services (Hall et al. 2019).

Engagement with outpatient care is crucial for preventing relapse and achieving full recovery (Doyle et al. 2014; Fridgen et al. 2013; Gearing et al. 2018). Disengagement (loss to follow‐up) in outpatient mental health care increases the risks of relapse, as the patients lack the opportunity to refill their medication and get advice on the importance of drug adherence. Poor drug adherence among patients with FEP is the major risk factor for relapse, with non‐adherence associated with exceeding a 90% risk of relapse and with approximately 30% of relapses occurring within 1 year after experiencing psychosis (McCann et al. 2011; Burns and Esterhuizen 2008; Jordan et al. 2018; Charlson et al. 2018; Alvarez‐Jimenez et al. 2012; Boydell et al. 2004).

A systematic review details the mental health service engagement among underserved minority adolescents and young adults and the pathways to mental health care of FEP patients (Moore 2018). This review exclusively considers intervention studies aiming to enhance mental health treatment for racial and ethnic minority groups. Another systematic review details rates of predictors of disengagement and strength of engagement for people with a FEP using Early Intervention Services (Robson and Greenwood 2022). This review concentrates solely on the engagement in early intervention services for young patients experiencing their first episode of psychosis, excluding regular outpatient mental health care. Additionally, the disengagement rate presented in this review may not be actual, as most inclusive studies report proportions, and the proportion of re‐engagement was not assessed.

Furthermore, the available review of the literature conceptualises the definition of service engagement within mental health services (Lal and Malla 2015). This scoping review encompassed regular outpatient mental health services and re‐engagement proportion; aspects not explored in the previous studies. Additionally, the review emphasises the adolescent and young adult age group, which is the primary age range at risk for disengagement. There are few reviews on the engagement, disengagement, and re‐engagement in outpatient mental health services among young patients with FEP in high‐income countries (HIC) and low‐ and middle‐income countries (LMICs). Therefore, this scoping review aims to map the evidence of the proportion of engagement, disengagement, and re‐engagement and identify their determinants to help identify barriers and inform facilitators for service engagement among FEP.

## 2 Methods

### 2.1 Search Strategy

This scoping review follows the guidelines for a scoping review by the Joanna Briggs Institute (JBI) and the Arksey and O'Malley framework (Peters et al. 2015; Arksey and O'Malley 2005). We follow the proposed steps for conducting a scoping review by identifying the review questions and relevant studies, selecting a systematic study, charting the data and then collating, summarising and reporting. We conducted the numerical and thematic analysis and reporting using the Preferred Reporting Items for Systematic Reviews and Meta‐Analysis Extension for scoping review (PRISMA‐ScR).

#### 2.1.1 Review Objectives

The following objectives guided this review.

1. To establish the proportion of young people with FEP that engage in outpatient mental health care.
2. To establish the proportion of young people with FEP that disengage from outpatient mental health care.
3. To establish the proportion of young people with FEP that re‐engage in outpatient mental health care following initial disengagement.
4. To establish the determinants of engagement, disengagement, and re‐engagement in outpatient mental health care among young patients with FEP.

#### 2.1.2 Definitions of Concept

Engagement is the ongoing commitment to receiving planned follow‐up mental health care services from mental health providers (Cowan et al. 2020).

Disengagement is the early discontinuation or withdrawal from mental health services before the planned course of treatment is completed (Doyle et al. 2014; Kim et al. 2019).

Re‐engagement is re‐establishing contact and resuming mental health care services after disengagement or dropout (Kim et al. 2019).

#### 2.1.3 Eligibility Criteria

This scoping review entailed using the Population, Concept and Context (PCC) mnemonic (Munn et al. 2020) to determine the study's eligibility based on this review's objectives (using Data S1). We included a study published in English from 1990, the expansion of psychiatric outpatient care (Kessler et al. 1999), which focused on engaged, disengaged and re‐engaged in outpatient mental health care using quantitative, qualitative, and mixed methods studies involving young patients with FEP aged 13–35 years. The inclusion of this age group is based on the existing literature (Lal and Malla 2015), suggesting that adolescents and young adults are prone to facing difficulties in engaging with mental health services. We included two studies that incorporated participants above the age of 35. One study provided a sub‐analysis comparing younger and older adults, while the other study, although it included participants aged 13–40, reported a mean participant age of 29.

#### 2.1.4 Information Sources

We searched for relevant studies, including PubMed, Web of Science and PsycINFO databases (Medline, CINAHL, Ebsco‐Host), and we also searched for unpublished reports on Google and World Health Organization (WHO) websites. The search was conducted from 05 September 2022 to 29 December 2022. The three independent reviewers manually checked all the reference lists in the selected articles, and no article was included after the initial search.

#### 2.1.5 Searching Strategies

We developed a comprehensive search strategy with the help of the subject librarian using a combination of keywords **Psychosis** OR “Psychotic Disorders” [Mesh] AND Engagement OR “Continuity of Patient Care” [Majr] OR “Patient Compliance” [Majr] OR Disengagement OR “Patient Dropouts” [Majr] OR Re‐engagement AND Young patients OR “Adolescent” [Majr] OR “Adult” [Majr] OR “Young Adult” [Mesh] AND First episode psychosis. (Data S2).

#### 2.1.6 Selection of the Study

Citations were exported to the reference manager, EndNote, and checked for duplicates. To ensure a systematic study selection, we used PRISMA‐ScR (Tricco et al. 2018).

##### 2.1.6.1 Title Review

Three independent reviewers screened the title, guided by the inclusion and exclusion criteria, to ensure trustworthiness; duplicates were checked and removed. Articles meeting the inclusion criteria were moved for abstract screening.

##### 2.1.6.2 Abstract Review

Three independent reviewers screened the abstract and sought a third opinion in case of disagreement, leaving articles for full‐text review.

##### 2.1.6.3 Full‐Text Review

**FIGURE 1:**
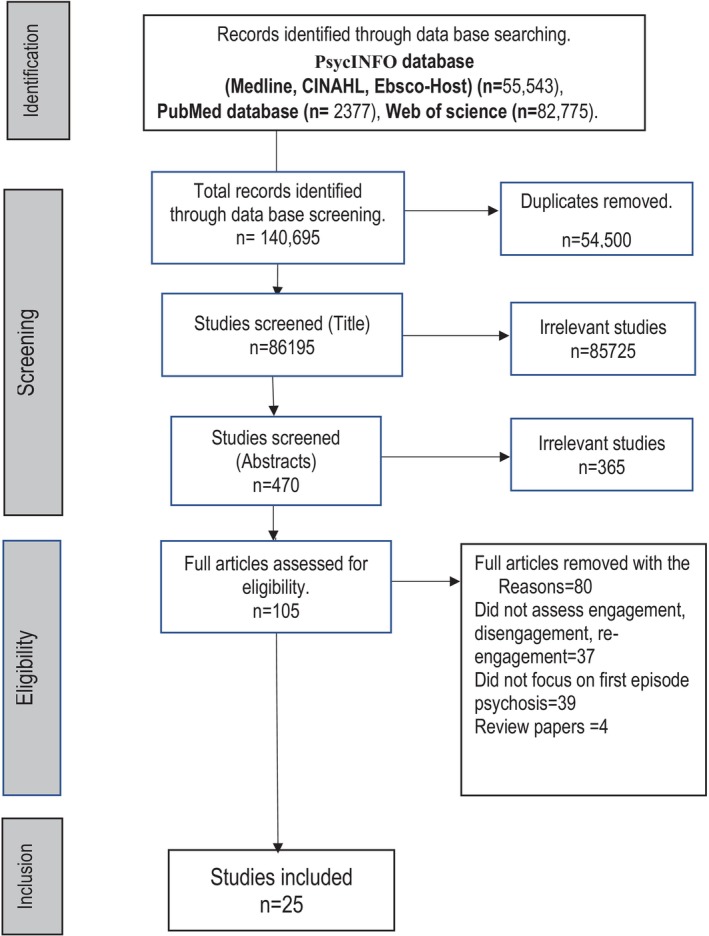
PRISMA flow diagram of the scoping review process.

The three independent reviewers completed the full‐text review, and articles meeting the inclusion criteria were retained for inclusion in this review. The PRISMA flow diagram was used to report the screening results (Figure 1).

#### 2.1.7 Methodological Quality Appraisal

We appraised the methodological quality of the included studies using the appropriate Critical Appraisal Skills Programme (CASP), specifically for cohort and qualitative studies (Buccheri and Sharifi 2017). We applied the JBI (Munn et al. 2020) appraisal tools for descriptive case series and cross‐sectional studies.

Two reviewers independently assessed each study, resolving discrepancies through consensus. The appraisal process informed our interpretation of the findings but did not lead to the exclusion of studies, as quality assessment is not a requirement in a scoping review.

#### 2.1.8 Charting the Data

We developed the charting table based on predefined criteria and extracted the information from all included studies according to the objectives of this review using the data extraction form (Data S1), the summary being presented in Table 1. One reviewer extracted the data, and the next independently verified the information extracted.

**TABLE 1:** Articles included in the scoping review.

Authors and year	Title	Study design and follow‐up time	Study location, country (continent)	Study settings	Age of the study participants (years)	Definition of FEP	Definition of engagement, disengagement, re‐engagement	Proportion, engagement*, disengagement**, re‐engagement***	Determinants
NovakGrubic and Tavcar 2002	Predictors of Noncompliance in Males With First‐Episode Schizophrenia, Schizophreniform, and Schizoaffective Disorder.	Cohort 12‐month follow‐up.	Ljubljana, Slovenia (Europe)	University Psychiatric Hospital in Ljubljana, Slovenia.	20–34	Not defined	Not defined	53.6%**	Lack of insight *x* ^2^ = 18.31, < 0.0001 PANSS positive *x* ^2^ = 5.31, 0.02 Diagnosis ×2 = 4.12, 0.04
Compton (2005)	Barriers to Initial Outpatient Treatment Engagement Following First Hospitalisation for a First Episode of Non‐affective Psychosis.	Descriptive Case series	Atlanta, USA (North America)	Urban Community Mental Health Centre, Atlanta, USA.	18–31	Not defined	Not defined	Not presented	Inadequate remission of paranoia. Impaired insight. Good family support. Involvement with criminal justice.
Schimmelmann et al. (2006)	Predictors of Service Disengagement in First‐Admitted Adolescents With Psychosis.	Cohort 18‐month follow‐up	Melbourne, Australia (Australia)	The Early Psychosis Prevention and Intervention Centre (EPPIC) in Melbourne, Australia.	15–29	Cohort receiving their first treatment in a specialised early prevention and intervention centre.	Actively refused any contact with the treatment facility or were not traceable	23.4%**	Living without family during treatment *x* ^2^ = 13.26, *p* < 0.001. Persistent SUD *x* ^2^ = 6.93, *p* = 0.022 Other psychoses *x* ^2^ = 0.21 *p* = 0.05
Turner et al. (2007)	Prediction of Twelve‐Month Service Disengagement From an Early Intervention in Psychosis Service.	Cohort 12‐month follow‐up time.	Christchurch, New Zealand (Australia)	A stand‐alone EIS in Christchurch, New Zealand	18–30	Defined as having consulted a medical professional for psychotic symptoms for the first time less than 2 years prior to entering the study	Termination of treatment despite therapeutic need within 12 months of entry.	24.6%**	long DUI < 0.05 PNSS < 0.005 Substance abuse < 0.01
Lecomte et al. (2008)	Predictors and Profiles of Treatment Non‐Adherence and Engagement in Services Problems in Early Psychosis	Cross‐sectional	Vancouver, Canada (North America)	South Fraser Early Psychosis Intervention (EPI) Program, the Vancouver EPI Program, the University of British Columbia Day program and St‐Paul's Hospital's Out‐Patient Clinic, Vancouver, Canada.	18–34	Having consulted a medical professional for psychotic symptoms for the first time less than 2 years prior to entering the study	SES		Physical abuse *β* = 0.34, *p* = 0.001. Alliances with a therapist (*β* = −0.28, *p b* 0.01), Knowledge consumer rights (*β* = −0.21, *p b* 0.05)
Miller et al. (2009)	A Prospective Study of Cannabis Use as a Risk Factor for Non‐Adherence and Treatment Dropout in First‐Episode Schizophrenia.	Cohort 12‐month follow‐up time	New York, USA (North America)	Preventing Morbidity in First Episode Schizophrenia Study, New York.	16–35	Not defined	Dropping out of treatment for more than 1 month and not following up with treatment elsewhere	20.53%**	Substance use (HR 6.4), (1.2, 35.6, *p* ≤ 0.05)
Turner et al. (2009)	Outcomes for 236 Patients From a 2‐Year Early Intervention in Psychosis Service.	Cohort 24‐month follow‐up time.	Christchurch, New Zealand (Australia)	A stand‐alone EIS in Christchurch, New Zealand.	18–30	Defined as having consulted a medical professional for the first time less than 2 years prior to entering the study.	Termination of treatment despite therapeutic need within 12 months of entry.	34%**	Unemployment, *p* < 0.01. GAF, *p* < 0.05
Conus et al. (2010)	Rate and Predictors of Service Disengagement in an Epidemiological First‐Episode Psychosis Cohort.	Cohort 18‐month follow‐up time.	Australia (Australia)	The Early Psychosis Prevention and Intervention Centre (EPPIC) in Australia.	15–29.	Not defined	Patients actively refused any contact with the treatment facility, or were not traceable	23.3%**	Forensic history *χ* ^2^ 15.5 *p* < 0.001 No work/school *χ* ^2^ = 4.4, *p* = 0.035 Living without family *χ* ^2^ = 13.2 *p* ≤ 0.001 Other psychosis *χ* ^2^ 4 *p* = 0.04 SUD HR = 2.30; 95% CI 1.45–3.66
Stowkowy et al. (2012)	Predictors of Disengagement From Treatment in an Early Psychosis Program.	Cohort 36‐month of follow‐up	Alberta, Canada (North America)	Calgary Early Psychosis Treatment Service (EPTS), Calgary, Alberta, Canada.	15–30	Not defined	Leaving the program before the end of 30 months	31%**	Lower negative symptoms score. (*t* [258] = −2.06, *p* = 0.05) No family member *χ* ^2^ = 20.92 df = 1 *p* = 0.0001. Shorter duration of untreated psychosis. *χ* ^2^ = 5.99 *p* = 0.014
Macbeth et al. (2013)	Service Engagement in First Episode Psychosis: Clinical and Premorbid Correlates.	Cross‐sectional	Scottish cities (Europe)	Early intervention psychosis services in two Scottish cities.	13–35	In the first 12 months of treatment for FEP	SES scale		PANSS Negative Symptoms *Β* = 31.4 *t* = 3.92 *p* < 0.001 Sex *t* = −3.552 *p* = 0.001
Anderson et al. (2013)	Determinants of Negative Pathways to Care and Their Impact on Service Disengagement in First‐Episode Psychosis.	Cohort 36‐ month of follow‐up	Montre ´al, Canada (North America)	Prevention and Early Intervention for Psychoses Program (PEPP) in Montre ´al, Canada.	14–30	Had ≤ 30 consecutive days of an antipsychotic drug therapy	No contact for a continuous period of 3 months	28% **	Older age (HR = 1.10, 1.02–1.19), Black patients (HR = 2.10, 1.19–3.70) Living alone, (HR = 0.46, 0.21–1.00)
Stewart (2013)	Factors Contributing to Engagement During the Initial Stages of Treatment for Psychosis.	Qualitative Grounded theory approach.	Australia (Australia)	Early psychosis program (EPP) located in an eastern city in Australia.	18–20	Not defined	Not defined		Nature of relationships among young patients and care providers. Transition between initial treatment and community care.
Zheng et al. (2013)	Rate and Predictors of Service Disengagement Among Patients With First‐Episode Psychosis.	Cohort 24‐month Follow‐up Time.	Singapore (Asia)	EPIP between 2001 and 2009, with 2 years of follow‐up data. Singapore	15–35	Not defined	Dropout after 2 years	14**	DUI = OR = 1.01 (1.00–1.01) *p* = 0.030 Malay ethnicity OR = 1.93, CI 1.12–3.29, *p* = 0.017 Education OR = 2.30, (CI 1.23–4.29) *p* = 0.009 Unemployment OR = 1.26, 1.01–1.97 *p* = 0.03
Chan et al. (2014)	Rate and Predictors of Disengagement From a 2‐Year Early Intervention Program for Psychosis in Hong Kong	Cohort 24‐month follow‐up time.	Hong Kong (Asia)	The EASY program in Hong Kong	15–25	Not defined	Continuous default of the EASY outpatient appointments up till the end of the 2‐year service despite, therapeutic need, and active tracing from staff for psychiatric follow‐up	0.05** at 6 months 0.09** at the end of the first year 0.13** at the end of the second year.	Age = *χ* ^2^ 0.16 *p* = 0.69 Gender *χ* ^2^ = 0.41 *p* = 0.52 Schizophrenia spectrum *χ* ^2^ = 17.98, *p* ≤ 0.001 Medication compliance *χ* ^2^ = 14.29 *p* ≤ 0.001 Substance abuse *χ* ^2^ = 7.94 *p* = 0.003.
Ouellet‐Plamondon et al. (2015)	Engaging Immigrants in Early Psychosis Treatment: A Clinical Challenge	Cohort 24‐month follow‐up time.	Montreal, Canada (North America)	5‐year early intervention programs in urban catchment areas of Montreal, Canada	18–30	Not defined	Not defined		No association
Casey et al. (2016)	Predictors of Engagement in First‐Episode Psychosis	Cohort 24‐ month of follow‐up	Birmingham, UK (Europe)	Patients were recruited from Birmingham and Solihull Mental Health NHS Foundation Trust (BSMHFT) early intervention on services over a 2‐year period, UK.	14–35	Not defined	Singh‐O'Brien Level of Engagement Scale (SOLES)^E^		SOLES SCORE Gender (*t* (103) = −0.21, *p* = 0.83) ethnicity (*F* [4] = 2.63, *p* = 0.054) No qualification (*F* [4] = 4.06, *p* < 0.009) Age (Pearson's *ρ* = 0.118, *p* = 0.236) Living with others (*F* [103] = −2.17, *p* = 0.032) Socio economic status (*F* [103] = 0.937, *p* = 0.43) Marital status (*F* [103] = −2.17, *p* = 0.032)
Myers et al. (2017)	Clinical Correlates of Initial Treatment Disengagement in First‐Episode Psychosis	Cross‐sectional	New York, USA (North America)	First‐episode patients were in usual treatment (i.e., the public mental health system team), New York, USA	18–21	Not defined	Attendance at three or more follow‐up appointments after an initial hospitalisation (3)	54.5%** 45.5%*	Medication adherence *z* = 3.46, *p* = 0.001. Knowledge about psychosis *T* = 3.16, *p* = 0.04 Insight = *t* = 2.39 *p* = 0.2 3 Education level = *t* = 2.07, df = 31, *p* = 0.047 Below federal poverty = *χ* ^2^ = 2.07 df = 1, Fisher's exact *p* = 0.05 Substance use *χ* ^2^ = 3.92, df = 1 *p* = 0.05
Nikolitch et al. (2018)	Adherence to Follow‐Up in First‐Episode Psychosis: Ethnicity Factors and Case Manager Perceptions.	Qualitative& quantitative	Montreal, Canada (North America)	The First Episode Psychosis Program (FEPP) at the Jewish General Hospital in Montreal, Canada	12–35		Good/bad adherence	50%**	Black, χ2 = 5.4, df = 1, *p* = 0.02
Maraj et al. (2018)	Disengagement in Immigrant Groups Receiving Services for a First Episode of Psychosis.	Cohort 24‐month follow‐up	Montreal, Canada (North America)	PEPP‐Montréal between January 2003 and July 2012, Montreal, Canada	14–35	Not defined	No clinical contact for at least three consecutive months	24.2%**	Age *p* = 0.04 Education level *p* = 0.041 Medication adherence *p* = 0.0001
Kim et al. (2019)	The Rates and Determinants of Disengagement and Subsequent Re‐Engagement in Young People With First‐Episode Psychosis	Cohort 24‐month follow‐up time	Melbourne, Australia (Australia)	Origen Youth Health in the North‐Western area of Melbourne, Australia	15–24	Not defined	Patients actively refused any contact with the treatment facility or were not traceable	56.3%** 78.8***	Age (HR = 1.24, 95% CI 1.01–1.51, *p* = 0.04) Not being in employment, education, or training (NEET) (HR = 1.76, 95% CI 1.44–2.15, *p* < 0.001). Psychiatry history in the family (HR = 0.71, 95% CI 0.54–0.92, *p* = 0.01) Substance abuse (HR = 1.38, 95% CI 1.24–1.54, *p* < 0.001). No factors were associated with re‐engagement.
Reynolds et al. (2019)	The association Between Community and Service Level Factors and Rates of Disengagement in Individuals With First‐Episode Psychosis.	Cohort 24‐month follow‐up time	Melbourne, Australia (Australia)	Early Psychosis Prevention and the Intervention Centre (EPPIC) service in Melbourne, Australia	15–24		Patients actively refused any contact with the treatment facility. or were not traceable	55.7%**	Social deprivation (HR = 1.047, 95% CI. 1.01–1.09, *p* = 0.014) NEET = (HR = 1.044, 95% CI 1.01–1.08, *p* = 0.022)
Tindall et al. (2019)	The Missing Voice of Engagement: An Exploratory Study From the Perspectives of Case Managers at an Early Intervention Service for First‐Episode Psychosis.	Qualitative study	Melbourne, Australia (Australia)	EIS in Melbourne Australia, between July 2016 and March 2017	15–25	Not defined	The case manager views perspectives on reasons for disengagement and engagement.		Enjoyment from working together. Mismatch service. Building trust and connection. Therapeutic alliances.
Lau et al. (2019)	Rates and Predictors of Disengagement of Patients With First‐Episode Psychosis From the Early Intervention Service for Psychosis Service (EASY) Covering 15 to 64 years of Age in Hong Kong.	Cohort 36‐month follow‐up, retrospectively.	Hong Kong (Asia)	The early intervention service (EASY) in Hong Kong.	15–25 26–64	Not defined	Loss to follow‐up over 3 months of follow‐up.	17.2%**	Drug compliance *x* ^2^ = 17.24,0.00 History of substance abuse *x* ^2^ = 6.34,0.01
Tindall et al. (2020)	Disengagement Processes Within an Early Intervention Service for First‐Episode Psychosis: A Longitudinal, Qualitative, Multi‐Perspective Study.	Qualitative	Melbourne, Australia (Australia)	Melbourne, Australia.	15–25	Not defined	Not defined		Service mismatch. A lack of shared purpose responses to individual circumstances
Golay et al. (2020)	Rates and Predictors of Disengagement Settings: Treatment and Early Intervention in Psychosis Program.	Cohort 36‐month follow‐up time	Lausanne, Switzerland (Europe)	Lausanne, Switzerland	18–35	Not defined	Actively refused despite active and repeated attempts.	15.5%**	Low socio‐economic status

## 3 Results

### 3.1 Characteristics of the Source of the Evidence

We retrieved 140 695 articles, screened 86 195 by title and screened 105 in full text. Twenty‐five articles met this review's inclusion criteria, with the study selection summary being presented using the PRISMA flow diagram (Figure 1). The overall methodological quality of the included studies was high, as most items in the appraisal checklist were addressed (Data S3). These studies were conducted in HIC from four continents: Europe, Australia, North America and Asia (Table 1). Most studies were conducted from early intervention services for FEP (Kim et al. 2019; Miller et al. 2009; Anderson et al. 2013; Maraj et al. 2018; Lecomte et al. 2008; Stowkowy et al. 2012; Casey et al. 2016; Schimmelmann et al. 2006; Chan et al. 2014; Conus et al. 2010; Macbeth et al. 2013; Reynolds et al. 2019; NovakGrubic and Tavcar 2002; Ouellet‐Plamondon et al. 2015; Turner et al. 2007, 2009; Tindall et al. 2020, 2019; Nikolitch et al. 2018; Stewart 2013; Golay et al. 2020; Zheng et al. 2013; Lau et al. 2019), with only two reporting the findings from usually outpatient mental health services (Compton 2005; Myers et al. 2017). Of the 25 articles, 24 reported disengagement and engagement (Miller et al. 2009; Anderson et al. 2013; Maraj et al. 2018; Lecomte et al. 2008; Stowkowy et al. 2012; Casey et al. 2016; Schimmelmann et al. 2006; Chan et al. 2014; Conus et al. 2010; Macbeth et al. 2013; Reynolds et al. 2019; NovakGrubic and Tavcar 2002; Ouellet‐Plamondon et al. 2015; Turner et al. 2007; Tindall et al. 2020; Tindall et al. 2019; Nikolitch et al. 2018; Stewart 2013; Turner et al. 2009; Golay et al. 2020; Zheng et al. 2013; Lau et al. 2019; Compton 2005; Myers et al. 2017), and one reported disengagement and re‐engagement (Kim et al. 2019). The majority of articles used a longitudinal cohort design (Kim et al. 2019; Miller et al. 2009; Anderson et al. 2013; Maraj et al. 2018; Lecomte et al. 2008; Stowkowy et al. 2012; Casey et al. 2016; Schimmelmann et al. 2006; Chan et al. 2014; Conus et al. 2010; Macbeth et al. 2013; Reynolds et al. 2019; NovakGrubic and Tavcar 2002; Ouellet‐Plamondon et al. 2015; Turner et al. 2007; Stewart 2013; Turner et al. 2009; Golay et al. 2020; Zheng et al. 2013; Lau et al. 2019), four used a qualitative approach (Tindall et al. 2020, 2019; Nikolitch et al. 2018; Stewart 2013), one used a descriptive case series (Compton 2005) and one used a cross‐sectional study design (Myers et al. 2017). Overall, 20 reported the methods used to measure engagement, disengagement, and re‐engagement (Kim et al. 2019; Miller et al. 2009; Anderson et al. 2013; Maraj et al. 2018; Lecomte et al. 2008; Stowkowy et al. 2012; Casey et al. 2016; Schimmelmann et al. 2006; Chan et al. 2014; Conus et al. 2010; Macbeth et al. 2013; Reynolds et al. 2019; Turner et al. 2007; Tindall et al. 2019; Nikolitch et al. 2018; Turner et al. 2009; Golay et al. 2020; Zheng et al. 2013; Lau et al. 2019; Myers et al. 2017), whereas five articles did not report the measures to assess engagement and disengagement (NovakGrubic and Tavcar 2002; Ouellet‐Plamondon et al. 2015; Tindall et al. 2020; Stewart 2013; Compton 2005), and most used dropout and attendance as proxy measures for disengagement and engagement.

Disengagement was measured differently across the studies: loss to follow‐up for 1 month (Miller et al. 2009), no contact for 2–3 months (Anderson et al. 2013; Maraj et al. 2018), missing more than three appointments consecutively (Myers et al. 2017), loss to follow up for 12 months (Turner et al. 2007), dropout after 2 years (Chan et al. 2014; Turner et al. 2009; Zheng et al. 2013), dropout after 3 years (Lau et al. 2019) good or bad attendance (Nikolitch et al. 2018), leaving the program before the end of 30 months (Stowkowy et al. 2012), case manager perception on engagement and disengagement (Tindall et al. 2019) and active refusal of any contact (Kim et al. 2019; Schimmelmann et al. 2006; Conus et al. 2010; Reynolds et al. 2019; Golay et al. 2020). In measuring engagement, one study used missing less than three scheduled visits in 6 months of follow‐up (Myers et al. 2017), while others used the Service Engagement Scale (SES) rated by the clinician (Lecomte et al. 2008; Macbeth et al. 2013) and the Singh O'Brien level of Engagement Scale (SOLES) (Casey et al. 2016). Both SES and SOLES measure the level of engagement of individuals in mental health services. However, SES measures service engagement based on clinician rate, while SOLES is a specific patient‐rated scale. The measure of re‐engagement used was face‐to‐face attendance after disengagement (Kim et al. 2019).

### 3.2 Synthesis of Evidence

The disengagement, engagement and re‐engagement are reviewed with respect to their proportions and determinants.

### 3.3 Proportion and Determinants of Disengagement

The disengagement proportion reported in this review ranges from 13% to 56.3% over a follow‐up period of 12 to 36 months (Table 1).

Socio‐demographic and related factors and clinical factors associated with disengagement were assessed.

### 3.4 Socio‐Demographic and Related Determinants of Disengagement

#### 3.4.1 Age

Eight articles assessed age as a determinant of disengagement, with inconsistent results. Three articles reported a significant association between an increase in age and the likelihood of disengagement (Kim et al. 2019; Anderson et al. 2013; Maraj et al. 2018), while others found no association (Stowkowy et al. 2012; Chan et al. 2014; Turner et al. 2007; Nikolitch et al. 2018; Lau et al. 2019). All studies used the same study designs; however, the studies that found associations also employed the same definition of disengagement.

#### 3.4.2 Gender

Eight articles assessed the relationship between gender and disengagement and found no association (Kim et al. 2019; Maraj et al. 2018; Lecomte et al. 2008; Stowkowy et al. 2012; Chan et al. 2014; Conus et al. 2010; Turner et al. 2007; Lau et al. 2019). The lack of association might be attributed to the minor differences in the proportion of male and female participants in these studies.

#### 3.4.3 Ethnicity

Very few studies compared ethnic groups and the level of disengagement in this review. Two studies that included multiple ethnic groups reported that Black patients are more likely to disengage than others (Anderson et al. 2013; Nikolitch et al. 2018), with one noting that Malay ethnicity predicted disengagement (Zheng et al. 2013). Both studies (Anderson et al. 2013; Nikolitch et al. 2018) were conducted in Canada, with the Black ethnicity representing a smaller proportion of the sample; however, the Black ethnicity was still twice as likely to disengage.

#### 3.4.4 Level of Education

Twelve articles reported the relationship between the level of education and disengagement. Three articles reported that not being educated and lacking job training increase the likelihood of disengagement compared to being educated and in training. Both studies (Kim et al. 2019; Reynolds et al. 2019) were conducted in Australia, and education levels were assessed categorically, considering factors such as lack of education, unemployment, and the absence of training, often referred to as NEET. Additionally, the studies conducted in the UK (Myers et al. 2017) and Singapore (Zheng et al. 2013) involved typical mental health services, not early intervention services. The lack of intervention in this study would contribute to this observed difference because no specialised follow‐up procedures were employed for the patients. Eight studies did not find a relationship between the level of education and the likelihood of disengagement (Anderson et al. 2013; Maraj et al. 2018; Stowkowy et al. 2012; Chan et al. 2014; Conus et al. 2010; Turner et al. 2007; Nikolitch et al. 2018; Lau et al. 2019); these studies used different study designs.

#### 3.4.5 Living Status

Two articles reported that patients living alone without family support were more likely to disengage (Schimmelmann et al. 2006; Conus et al. 2010). These two studies used the same measure of disengagement and were conducted in Australia, suggesting a possible culture emphasising family support. Similarly, (Stowkowy et al. 2012) found that those with no family in the program were more likely to disengage, with 72% of participants having family involvement. However, one study noted a negative association between patients living with their family and being more likely to disengage than those living without the family (Anderson et al. 2013); in this study, about 17.9% of the sample had missing information about the living arrangement, and another did not find a relationship between living status and the level of disengagement (Maraj et al. 2018).

#### 3.4.6 Employment Status

Four studies reported that not being at work or school increases the likelihood of disengagement (Kim et al. 2019; Turner et al. 2007, 2009; Zheng et al. 2013). Three studies were done in Australia and one in Singapore (Zheng et al. 2013), but most of the sample was unemployed.

#### 3.4.7 Poverty and Social Deprivation

One article reported social deprivation as a significant factor in disengagement (Reynolds et al. 2019). This is the only study that assesses social deprivation as a risk of disengagement. The evidence shows that the services located in the socially deprived areas are also under‐resourced to manage the needs of the people, which probably increases the risk of disengagement. Additionally, another study reveals the association between the increased likelihood of disengagement among patients living below the federal poverty level (Myers et al. 2017). This study was done in the usual mental health services, and the participants needed support to cater to their needs; therefore, those living below the poverty line were more likely to disengage.

#### 3.4.8 Substance Use

Most articles reported that the persistent use of cannabis and alcohol increases the risk of disengagement (Kim et al. 2019; Miller et al. 2009; Schimmelmann et al. 2006; Chan et al. 2014; Conus et al. 2010; Turner et al. 2007; Lau et al. 2019), with a few finding no significance (Anderson et al. 2013; Maraj et al. 2018; Ouellet‐Plamondon et al. 2015). In the studies that found no association, there is a slight difference in the proportion of participants between those who used the substance and those who did not.

#### 3.4.9 Involvement With Criminal Justice

Three articles reported that patients with a criminal justice/history of criminal records were more likely to disengage (Conus et al. 2010; Golay et al. 2020; Compton 2005). Although these studies were done across countries and used different designs, they reveal the same conclusion.

### 3.5 Clinical Determinants of Disengagement

#### 3.5.1 Medication Adherence

Four articles found an association between medication adherence and disengagement; those with poor medication adherence were more likely to disengage (Maraj et al. 2018; Chan et al. 2014; Lau et al. 2019; Myers et al. 2017). These studies were conducted on different continents and utilised different study designs.

#### 3.5.2 Knowledge About Psychosis and Insight

One article reported that knowledge about mental health consumer rights/psychiatric patients' rights increases the likelihood of disengagement (Lecomte et al. 2008).

#### 3.5.3 Positive and Negative Syndrome Scale

The Positive and Negative Syndrome Scale (PANSS) is a clinical tool designed to evaluate the severity of symptoms in individuals with schizophrenia. It measures both positive symptoms, like hallucinations and delusions, and negative symptoms, such as social withdrawal and lack of motivation (Lindenmayer et al. 2004). Most articles reported that patients with lower positive and negative syndrome scores were more likely to disengage from the services (Stowkowy et al. 2012; Macbeth et al. 2013; NovakGrubic and Tavcar 2002; Turner et al. 2007), while two reported no association between them (Zheng et al. 2013; Myers et al. 2017). In this study, the positive and negative symptom scores did not differ among the groups at baseline.

#### 3.5.4 Duration of Untreated Psychosis

Four studies reported that patients with a shorter duration of untreated psychosis (DUP) were more likely to disengage than those with a longer DUP (Stowkowy et al. 2012; Turner et al. 2007, 2009; Zheng et al. 2013).

#### 3.5.5 History of Mental Illness in the Family

One article reported that patients with a first‐degree relative with a psychotic disorder were less likely to disengage (Kim et al. 2019) compared to families without a history of mental illness.

#### 3.5.6 Psychosis Diagnosis and Other Symptoms

Three studies compared the type of diagnosis and the risk of disengagement and found that patients with psychotic disorders other than schizophrenia‐spectrum disorders (acute and transient psychosis), unspecified non‐organic psychosis and affective disorders with psychotic features were more likely to disengage (Schimmelmann et al. 2006; Chan et al. 2014; Conus et al. 2010). One study noted that patients with inadequate remission and poor insight, lower premorbid functioning, shorter duration of prodrome and lower global function score were more likely to disengage (Conus et al. 2010). Two articles report that a higher Global Assessment of Functioning (GAF) score increased the likelihood of disengagement (Stowkowy et al. 2012; Ouellet‐Plamondon et al. 2015).

### 3.6 Proportion and Determinants of Engagement

Four articles report on factors associated with engagement, with three not indicating the proportion of engagement (Lecomte et al. 2008; Casey et al. 2016; Macbeth et al. 2013) and one noting a 45.5% level of engagement for 6 months among FEP (Myers et al. 2017). The socio‐demographic and clinical‐related determinants were assessed.

### 3.7 Socio‐Demographic and Related Determinants of Engagement

The eight socio‐demographic determinants of engagement reviewed were age, gender, ethnicity, level of education, marital status, living status, socio‐economic status and substance use.

#### 3.7.1 Age

Two articles reported on the relationship between age and the level of engagement; one used the SOLES score, and the other used SES as a measure of engagement, but neither found an association between engagement and differences in age (Casey et al. 2016; Macbeth et al. 2013).

#### 3.7.2 Gender

One article used the SOLES score to measure engagement, but the score did not differ between females and males (Casey et al. 2016). A study that used SES found females' gender to be more likely to engage in care than males (Macbeth et al. 2013). Both studies were done in Europe, but the study that found no significance used a clinician‐rated scale; the clinician's perception of male engagement in care probably could contribute to this difference.

#### 3.7.3 Ethnicity

A study that used SOLES to compare ethnic groups shows no relationship in the level of engagement (Casey et al. 2016). The ethnic groups' distribution in this study differs very slightly.

#### 3.7.4 Level of Education

One article that used SOLES reported that patients with low education or no qualifications were more likely to engage compared to other groups (Casey et al. 2016). However, another article that used attendance of more than three outpatients scheduled within 6 months of follow‐up found that patients with higher education levels were more likely to engage (Myers et al. 2017). Also, this study was conducted in the usual outpatient mental health services and not in the early intervention services.

#### 3.7.5 Marital Status

One study using SOLES found that married patients did not have a more significant engagement score compared to unmarried persons (Casey et al. 2016). This study showed a slight proportion difference between married and unmarried individuals.

#### 3.7.6 Living Status

One article reported that patients living with their families were more likely to engage in care (Casey et al. 2016).

#### 3.7.7 Socio‐Economic Status

The one article that used SOLES as a measure of engagement found no significant association between the socio‐economic group and the level of engagement (Casey et al. 2016).

#### 3.7.8 Substance Use

One article reported that the patients who engaged in care were less likely to have used drugs for the previous 6 months (Myers et al. 2017).

### 3.8 Clinical Determinants and Engagement

#### 3.8.1 Medication Adherence

One study that assessed medication adherence as a determinant for engagement found that patients with good adherence were more likely to engage in care than other groups (Myers et al. 2017).

#### 3.8.2 PANSS

One article reported that patients with more positive symptoms were more likely to engage in care (Myers et al. 2017). Another found that patients with poor clinician engagement rates were associated with higher positive and negative symptom scores and severity of symptoms (Macbeth et al. 2013).

#### 3.8.3 DUP

One study reported that patients with a low score in the DUP had high engagement scores (Casey et al. 2016).

#### 3.8.4 Psychotic Diagnosis and Other Symptoms

One study assessed the level of engagement and the type of diagnosis but found no significant relationship (Casey et al. 2016), while social premorbid adjustment, premorbid academic adjustment, and general assessment of functioning were not significantly associated with the engagement score. Significantly higher engagement scores were found in patients who strongly believed social stress was an important cause of FEP (Macbeth et al. 2013).

### 3.9 Proportion and Determinants of Re‐Engagement

Re‐engagement in mental health services helps individuals resume and continue their mental health treatment and support to improve their well‐being and manage their mental health conditions effectively after disengagement. One article reported on the level of re‐engagement. Overall, re‐engagement remained high, with 85.5% after the first disengagement, 79.1% following the second and 78.8% after the third, slightly declining across subsequent episodes but found no socio‐demographic or clinical factors associated with re‐entry into care (Kim et al. 2019).

## 4 Discussion

### 4.1 Summary of Evidence

Most articles featured in this review focus on studies in Early Psychosis Intervention Programs in HICs. There is a notable absence of research in LMICs and from Africa. This gap may be attributed to the limited presence of such programs in LMICs and a scarcity of published or evaluated research on this topic. The disengagement level reported in this review ranged from 13% to 56.3% over a 12–36‐month follow‐up period.

### 4.2 Socio‐Demographic and Related Determinants of Disengagement and Engagement

In this review, only three articles reported age as one of the determinants of disengagement (Kim et al. 2019; Anderson et al. 2013; Maraj et al. 2018), with older patients being more likely to disengage than younger ages. One probable explanation for this is that as age increases, people have increasing confidence in their ability to decide for themselves and rely less on the opinions of other family members, resulting in being more likely to disengage, irrespective of the consequences. Two articles reported the relationship between gender and the level of disengagement, with males being more likely to disengage than females (Anderson et al. 2013; Nikolitch et al. 2018). In most studies of health care utilisation and gender (Furimsky et al. 2008; Gearing et al. 2018), males are less likely to seek service than females, specifically during the early stages of health problems when they regard it as manageable or deny its existence and tend to wait until it becomes a problem. However, more men are diagnosed with substance use disorder (SUD) than women; this is an independent factor for disengagement, meaning that retention in care is particularly important for this population (Lev‐Ran et al. 2013). SUD impairs judgement and decision‐making abilities, leading individuals to prioritise other things over seeking medical help (van Toor et al. 2011).

A low level of education has also been reported as a determinant of service disengagement (Kim et al. 2019; Anderson et al. 2013; Conus et al. 2010; Reynolds et al. 2019; Myers et al. 2017), with a lack of insight into and understanding of health status having been reported as a determinant of low healthcare utilisation (Wei et al. 2015). In addition, being employed and attending school reduces the risk of disengagement (Anderson et al. 2013; Conus et al. 2010; Turner et al. 2009), with schools playing a key part in an individual's development in terms of benefitting peer relationships, social interactions, academic attainments, cognitive progress, emotional control, behavioural expectations, as well as physical and moral development (Bowman et al. 2017).

Although family support has been a critical factor in helping patients to remain in care, in this review, the unmarried were more likely to engage than the married (Casey et al. 2016). This may be due to most studies reporting on early intervention programmes, where clinicians provide assertive patient care and family members play a minimal role and are therefore not regarded as a factor, as even those living alone have access to help and support from the program. However, emerging evidence from recent studies conducted in Singapore and Italy, although not included in this review, indicates that family involvement is not universally beneficial in mental health care (Pelizza et al. 2024; Chua et al. 2024). These findings show that families can sometimes have a mixed or negative influence on the treatment process. Caregivers may actively discourage engagement with treatment, particularly the use of psychiatric medication, which can lead to reduced adherence and eventual disengagement from services. Also, patients living below the federal poverty line and those with social and material deprivation were more likely to disengage (Reynolds et al. 2019; Myers et al. 2017), with socio‐economic deprivation having been linked with longer DUP and negative pathways to care, which increased the risk for disengagement.

Involvement with criminal justice is reported as a risk of disengagement (Conus et al. 2010; Compton 2005); the evidence shows that individuals with a history of criminal justice are also subjected to other behaviours, which may increase the risk of disengagement. Ethnicity has also been reported as a factor associated with disengagement, with Black patients being more likely to disengage than other groups (Anderson et al. 2013; Nikolitch et al. 2018). While the relationship between disengagement and ethnic minority groups has been well reported, it is not known if the Black patients from these studies are from minority groups.

### 4.3 Clinical Determinants of Disengagement and Engagement

Poor medication adherence has been reported as a risk for disengagement, with studies having shown that patients who fail to engage with the treatment regimen are at higher risk of not continuing to follow up in care (Maraj et al. 2018; Chan et al. 2014; Myers et al. 2017). Poor medication adherence is associated (Peritogiannis et al. 2025) with broader challenges, as patients may feel that the treatment does not help, and the lack of trust in its effectiveness may result in disengagement. Other factors, such as medication side effects and financial challenges, have been associated with poor medication adherence, which may result in poor quality of life and lead to disengagement (Weiden and Buckley 2007). Emerging evidence underscores the importance of a person‐centred approach, which fosters trust, supports shared decision‐making and strengthens long‐term engagement and treatment adherence. In this context, the service user plays the “conductor” role, actively directing their care journey based on their unique needs, preferences and readiness for treatment (Ferrari et al. 2024). It involves balancing service user autonomy with provider control across various domains such as treatment, symptoms, relationships and life activities. It also emphasises the need to strengthen community‐based services, focusing on the role of Mobile Mental Health Units (MMHUs) in sustaining engagement and improving patient outcomes (Peritogiannis et al. 2025).

Patients with low positive and negative syndrome symptoms were more likely to disengage from care (Stowkowy et al. 2012; Schimmelmann et al. 2006; Chan et al. 2014; Turner et al. 2007; Myers et al. 2017), with studies showing that most do so after feelings of recovery and thinking that they do not need treatment anymore. Also, patients with a shorter DUP are linked with a positive pathway to care, which allows for the quick remission of symptoms; hence, the risk of disengagement (Turner et al. 2007).

In this review, patients with diagnoses other than schizophrenia were more likely to disengage (Schimmelmann et al. 2006; Chan et al. 2014; Conus et al. 2010). The evidence shows that these conditions usually have a severe course of illness and large impairment that requires treatment, making those affected less likely to disengage (Hamilton et al. 2019).

Finally, patients from a family with a history of mental illness were less likely to disengage (Kim et al. 2019), possibly due to the families being aware of the risk of disengagement and being able to control the treatment provided.

The difference in the level of disengagement presented in this review could be due to the study design and the definition of disengagement used in the study. The two studies, with low proportions, have different designs. One study excluded participants at risk of disengagement, namely those with SUD and a forensic history (Golay et al. 2020). The one in Melbourne, Australia, used the broader definition of disengagement: no contact for more than a year (Kim et al. 2019). One study with a high proportion found that 56.3% of patients disengaged based on a strict measure of less than three visits in 6 months of follow‐up (Myers et al. 2017), with other studies that used the same measure reporting similar proportions (Miller et al. 2009; Anderson et al. 2013; Maraj et al. 2018; Schimmelmann et al. 2006; Conus et al. 2010; Reynolds et al. 2019). Four studies reported on factors associated with engagement, with only one noting the actual proportion (Myers et al. 2017); two others used SES, and one that used SOLES did not report the proportion (Lecomte et al. 2008; Casey et al. 2016; Macbeth et al. 2013), while only one reported on re‐engagement (Kim et al. 2019).

The only paper that reported on re‐engagement did not refer to any associated factors that may have affected re‐entry to care.

### 4.4 Study Limitation

This review includes only the articles written in English, with the findings from studies reported in other languages being excluded, which might affect the review's scope. It also included reports from early intervention studies for FEP in HIC settings. However, multiple search strategies were used, and a wide range of studies was included to understand the topic better.

### 4.5 Future Directions

Given the high magnitude of disengagement in the FEP, intervention studies that reduce individual and clinical‐related risk factors for disengagement are highly required, with future studies needing to focus on factors associated with enabling early identification, preventing disengagement and encouraging re‐engagement, especially in Africa.

## 5 Conclusion

Although most of the articles in this review include reports from early intervention studies for FEP, the disengagement level is generally high, the differences among the studies being due to the absence of an internationally recognised standard definition of disengagement. The determinants for disengagement also varied among the studies, indicating the need for a growing body of knowledge to guide future studies, inform practical applications, and facilitate decision‐making processes. The findings suggest the need for healthcare providers to work closely with their patients to understand the factors for disengagement for persons with FEP and ensure they have the best possible quality of life.

## Conflicts of Interest

The authors declare no conflicts of interest.

## Supporting information


**Data S1:** eip70100‐sup‐0001‐supinfo_1.docx.

**Data S2:** eip70100‐sup‐0002‐supinfo_2.docx.

**Data S3:** Critical appraisal checklist for articles included in the scoping review.

## Data Availability

The authors have nothing to report.
