# Supplementary material for: Engagement, Disengagement and Re‐Engagement in Mental Health Services Among Young Patients With First‐Episode Psychosis: A Scoping Review
Source: Early Interv Psychiatry. 2025 Oct 9;19(10):e70100. doi: 10.1111/eip.70100 (PMC12509053; doi:10.1111/eip.70100)
Supplement: Supplementary file 1 — Data S1: eip70100‐sup‐0001‐supinfo_1.docx. [file EIP-19-0-s001.docx]

**Engagement, Disengagement and re-engagement in mental health services among young patients with First-episode psychosis. A scoping review of the quantitative, qualitative, and mixed methods evidence.**

Data extraction form.

Inclusion criteria

Study approach: qualitative, quantitative, and mixed methods design

Language: Studies published in English only.

The phenomenon of interest: Geographical: will include the study done in high-income and low- and middle-income countries.

Point of care: outpatient mental health services.

Time: Will search all the publications from 1990, the beginning of Psychiatric outpatient care.

Study details

Title_______

First Author___________

Publication date___________

Study approach: qualitative, quantitative, and mixed methods design

Study designs:

Study population: young patients with FEP 13 to 35 years.

Definition of FEP:

Measure of engagement

Measure of disengagement

Measure of re-engagement

Study contents.

The phenomenon of interest: Engaged, disengaged, and re-engaged in outpatient mental health services for FEP.

Data is based on the following.

- To establish the proportion of young people with FEP that engage in outpatient mental health care.
- To establish the proportion of young people with FEP that disengage from outpatient mental health care.
- To establish the proportion of young people with FEP that re-engage in outpatient mental health care following initial disengagement.
- To establish the determinants of engagement, disengagement, and re-engagement in outpatient mental health care among young patients with FEP.
