# Supplementary material for: Engagement, Disengagement and Re‐Engagement in Mental Health Services Among Young Patients With First‐Episode Psychosis: A Scoping Review
Source: Early Interv Psychiatry. 2025 Oct 9;19(10):e70100. doi: 10.1111/eip.70100 (PMC12509053; doi:10.1111/eip.70100)
Supplement: Supplementary file 2 — Data S2: eip70100‐sup‐0002‐supinfo_2.docx. [file EIP-19-0-s002.docx]

| 1 | Search: **psychosis** Filters: **from 1990 - 2022** | 68,573 |
| --- | --- | --- |
| 2 | "Psychotic Disorders"[Mesh] | 35,937 |
| 3 | 1 OR 2 | 68,573 |
| 4 | Search**: Engagement** Filters: **from 1990 - 2022** | 279,069 |
| 5 | "Continuity of Patient Care"[Majr] | 137,950 |
| 6 | "Patient Compliance"[Majr] | 36,886 |
| 7 | 4 OR 5 OR 6 | 443,596 |
| 8 | Disengagement | 6,983 |
| 9 | "Patient Dropouts"[Majr] | 2,581 |
| 10 | 8 OR 9 | 9,540 |
| 11 | Re-engagement | 367 |
| 12 | 7 OR 10 OR 11 | 450,457 |
| 13 | Young patients | 653,451 |
| 14 | "Adolescent"[Majr] | 2,242 |
| 15 | "Adult"[Majr] | 18,399 |
| 16 | "Young Adult"[Mesh] | 1,005,443 |
| 17 | 13 OR 14 OR 15 OR 16 | 1,181,910 |
| 18 | First episode psychosis | 7,482 |
| 19 | 3 AND 12 AND 17 AND 18 | 2,377 |

Search strategy: Pubmed, date of last search 29^th^ December 2022
