# Supplementary material for: Engagement, Disengagement and Re‐Engagement in Mental Health Services Among Young Patients With First‐Episode Psychosis: A Scoping Review
Source: Early Interv Psychiatry. 2025 Oct 9;19(10):e70100. doi: 10.1111/eip.70100 (PMC12509053; doi:10.1111/eip.70100)
Supplement: Supplementary file 3 — Data S3: Critical appraisal checklist for articles included in the scoping review. [file EIP-19-0-s003.docx]

**CASP critical appraisal checklist of studies Included in this Scoping Review**

|  | Are the results of the review valid | | | | | | | | What are rsults | | | Will results help locally? | | |  |
| --- | --- | --- | --- | --- | --- | --- | --- | --- | --- | --- | --- | --- | --- | --- | --- |
| Author and year | Did the study address a clearly focused issue? | Was the cohort recruited in an acceptable way | Was the exposure accurately measured to  minimise bias? | Was the outcome accurately measured to  minimise bias? | Have the authors identified all important  confounding factors? | Have they taken account of the  confounding factors in the design  and/or analysis? | Was the follow up of subjects complete  enough? | Was the follow up of subjects long  enough? | What are the results of this study? | How precise are the results? | Do you believe the results? | Can the results be applied to the local population? | Do the results of this study fit with other  available evidence? | What are the implications of this study for practice? | Total  score |
| Novak-Grubic, V et al (2002) | 2 | 2 | 1 | 2 | 2 | 2 | 2 | 1 | 2 | 1 | 2 | 2 | 2 | 2 | 25 |
| Schimmelmann, B.G. et a (2006) | 2 | 2 | 2 | 2 | 2 | 2 | 2 | 1 | 2 | 2 | 2 | 2 | 2 | 2 | 27 |
| Turner, M. et al.  (2007) | 2 | 2 | 2 | 2 | 2 | 2 | 2 | 2 | 2 | 2 | 2 | 2 | 2 | 2 | 28 |
| Lecomte, T et al  (2009) | 2 | 2 | 2 | 2 | 2 | 2 | 2 | 2 | 2 | 2 | 2 | 2 | 2 | 2 | 28 |
| Miller, R et al  (2009) | 2 | 2 | 2 | 2 | 1 | 1 | 2 | 2 | 2 | 2 | 2 | 2 | 2 | 2 | 26 |
| Turner, M  (2009) | 2 | 2 | 2 | 1 | 1 | 1 | 2 | 2 | 2 | 2 | 2 | 2 | 2 | 2 | 25 |
| Conus, P et al  (2010) | 2 | 2 | 2 | 2 | 1 | 1 | 2 | 2 | 2 | 2 | 2 | 2 | 2 | 2 | 26 |
| Stowkowy, J. et al  (2012) | 2 | 2 | 2 | 2 | 2 | 2 | 2 | 2 | 2 | 2 | 2 | 2 | 2 | 2 | 28 |
| Anderson, K. K. et al  (2013) | 2 | 2 | 2 | 2 | 2 | 2 | 2 | 2 | 2 | 2 | 2 | 2 | 2 | 2 | 28 |
| Zheng, S. et al  (2013) | 2 | 2 | 2 | 2 | 2 | 2 | 2 | 2 | 2 | 1 | 2 | 2 | 2 | 2 | 27 |
| Chan, T. C. W et al  (2014) | 2 | 2 | 2 | 2 | 2 | 2 | 2 | 2 | 2 | 2 | 2 | 2 | 2 | 2 | 28 |
| Ouellet-Plamondon, C et al (2015) | 2 | 2 | 2 | 2 | 2 | 2 | 2 | 2 | 2 | 2 | 2 | 2 | 2 | 2 | 28 |
| Casey, D. et al  (2016) | 2 | 2 | 2 | 2 | 2 | 2 | 2 | 2 | 2 | 2 | 2 | 2 | 2 | 2 | 28 |
| Maraj, A. et al  (2018) | 2 | 2 | 2 | 2 | 2 | 2 | 2 | 2 | 2 | 2 | 2 | 2 | 2 | 2 | 28 |
| Kim, D.J et al  (2019) | 2 | 2 | 2 | 2 | 2 | 2 | 2 | 2 | 2 | 2 | 2 | 2 | 2 | 2 | 28 |
| Reynolds, S et al  (2020) | 2 | 2 | 2 | 2 | 2 | 2 | 2 | 2 | 2 | 2 | 2 | 2 | 2 | 2 | 28 |
| Golay, P. et al (2020) | 2 | 2 | 2 | 2 | 2 | 2 | 2 | 2 | 2 | 2 | 2 | 2 | 2 | 2 | 28 |
| Lau, K.W. et al | 2 | 2 | 2 | 2 | 2 | 2 | 2 | 2 | 2 | 2 | 2 | 2 | 2 | 2 | 28 |

CASP critical score, criterion is completely met =2, criterion is partially met =1 criterion not applicable or not mentioned =0

Interpretation of the total score 20-28: High quality, 10-19 moderate quality, 0-9 low quality.
